# Higher Concentration of Plasma Glial Fibrillary Acidic Protein in Wilson Disease Patients with Neurological Manifestations

**DOI:** 10.1002/mds.28509

**Published:** 2021-01-27

**Authors:** Jie Lin, Yexiang Zheng, Ying Liu, Yi Lin, Qiqi Wang, Xiao‐Hong Lin, Wenli Zhu, Wei‐Hong Lin, Ning Wang, Wan‐Jin Chen, Ying Fu

**Affiliations:** ^1^ Department of Neurology and Institute of Neurology of First Affiliated Hospital Institute of Neuroscience, Fujian Medical University Fuzhou China; ^2^ Department of Radiology of First Affiliated Hospital Fujian Medical University Fuzhou China; ^3^ Fujian Key Laboratory of Molecular Neurology Fujian Medical University Fuzhou China

**Keywords:** biomarker, GFAP, Wilson disease

## Abstract

**Background:**

Wilson disease is a rare, disabling, neurological genetic disease. Biomarkers of brain damage are less well developed.

**Objective:**

The aim of this study was to evaluate the utility of plasma glial fibrillary acidic protein as a biomarker for neurological involvement in patients with Wilson disease.

**Methods:**

This prospective cross‐observational study compared plasma glial fibrillary acidic protein concentration among different subtypes of patients with Wilson disease and healthy control subjects. Plasma glial fibrillary acidic protein levels were measured in 94 patients and 25 healthy control subjects. Patients were divided into two subtypes: patients with neurological manifestations (n = 74) or hepatic manifestations (n = 20).

**Results:**

Median levels of plasma glial fibrillary acidic protein were significantly elevated in patients with neurological manifestations (143.87 pg/mL) compared with those with hepatic manifestations (107.50 pg/mL) and healthy control subjects (86.85 pg/mL). Receiver operating characteristic curve revealed that a plasma glial fibrillary acidic protein cutoff value of 128.8 pg/mL provides sufficient sensitivity (80.0%) and specificity (63.5%) to differentiate patients with neurological manifestations from those with hepatic manifestations.

**Conclusions:**

Plasma glial fibrillary acidic protein may serve as a biomarker for distinguishing different subtypes of Wilson disease. © 2021 The Authors. *Movement Disorders* published by Wiley Periodicals LLC on behalf of International Parkinson and Movement Disorder Society

Wilson disease (WD) is a rare autosomal recessive inherited disorder of copper metabolism, with a genetic prevalence of approximately 13.9 per 100,000 individuals.^1^, ^2^ The *ATP7B* gene, which is mapped to chromosome 13 (13q14.3), encodes a transmembrane copper‐transporting P‐type ATPase.^3^ Pathogenic mutations in *ATP7B* lead to copper overload in organs, predominantly the liver and the brain, resulting in the occurrence of hepatic and neurological manifestations.^4^ Neurological manifestations in WD commonly occur later, after hepatic symptoms, potentially because of secondary copper aggregation after excess copper is released into the circulation from the liver.^5^, ^6^

Although rare, WD can be managed successfully if recognized early, diagnosed accurately, and treated correctly.^7^ However, neurological impairments are often irreversible or incompletely reversible. It is therefore essential to identify and monitor nervous system damage caused by copper toxicity in patients. However, commonly used biochemical indicators, including 24‐hour urinary copper and serum non‐ceruloplasmin‐bound copper, do not correlate with WD subtypes or severity. A persistent lack of reliable biomarkers for neurological damage has prevented the development of informative monitoring indices.

Long‐term exposure to high copper concentrations ultimately results in damaged astrocytes and dysfunction of the blood–brain barrier.^8^, ^9^ Furthermore, previous studies have shown that abnormal astrocytes present in the lesions of WD patient pathology.^9^, ^10^, ^11^ Given these findings, circulating astrocytic injury markers could be of interest in patients with WD. Specifically, we hypothesized that the concentration of circulating glial fibrillary acidic protein (GFAP), a marker of astrocyte damage,^12^ would be elevated in patients with WD with neurological manifestations (WD‐NM).

A biomarker that clearly differentiates neurological from nonneurological WD would likely have substantial utility. For example, it could be used to predict prognosis or as a measure of relevant disease activity or target engagement in clinical trials. Hence we examined the levels of plasma GFAP (pGFAP) among patients with WD to investigate whether GFAP values were correlated with the presence of neurological involvement and disease severity.^13^, ^14^

## Patients and Methods

### Study Design

These genetically confirmed patients were recruited from a WD cohort study (A Registered Cohort Study on Wilson disease, ClinicalTrials.org: NCT04012658) at the Neurogenetic Diseases Center in the First Affiliated Hospital of Fujian Medical University and were consecutively enrolled between July 1, 2019, and June 1, 2020. A diagnosis of WD was established based on typical symptoms and clinical, biochemical, and genetic findings. A positive diagnosis for WD was determined by a Leipzig score of ≥4.^15^ All patients were examined in outpatient clinics. To exclude the confounding effects of neurological symptoms, all participants were required to be free of other disease. In total, 94 participants with WD were included in the study.

Patients were divided into two subgroups: WD‐NM, defined as the presence of typical neurological symptoms regardless of whether they were accompanied by hepatic abnormalities; and WD with hepatic manifestations (WD‐HM), defined as the absence of neurological symptoms and the presence of hepatic abnormalities, typically characterized by abnormal liver function test, diffuse lesions of the liver parenchyma observed in ultrasound tests, or symptoms of cirrhosis.

We further recruited 25 healthy control subjects (HCs; 11 male and 14 female subjects; age range, 14–73 years), all of whom appeared to have normal brain MRI results. The study protocol and informed consent procedures were approved by the institutional review boards and ethics committee at First Affiliated Hospital of Fujian Medical University. Written informed consent was provided by all participants.

### Procedures

Direct sequencing of all exons and flanking regions in the *ATP7B* gene were performed as previously reported.^16^ The variants were identified and classified according to American College of Medical Genetics Standards and Guidelines.^17^

All patients' medical records and brain MRI data were collected. An experienced neurologist collected demographic information and clinical characteristics by questionnaire, including age at onset, initial symptoms, disease duration, assistant examination, current medications, and Unified Wilson's Disease Rating Scale (UWDRS) at the same time as plasma collection. Neurological symptom severity was evaluated by UWDRS, including functional impairment (UWDRS‐F, 9‐item), neurological examination (UWDRS‐N, 18‐item), and psychiatric assessment (UWDRS‐P, 19‐item).^13^ The degree of brain parenchyma damage in patients with WD was quantified by severity scale for brain MRI, and the resulting severity scales were assessed by two blinded radiologists. Before scoring, both radiologists were provided with a published scoring manual with example images.^14^

Blood sampling was conducted by collecting 10 mL venous blood in EDTA‐containing tubes, followed by centrifugation at 3500*g* for 10 minutes at 20°C, with subsequent storage of plasma aliquots in cryogenic vials at −80°C until use. pGFAP levels were measured using a commercial Simoa GFAP Discovery Kit (Quanterix, Lexington, MA, USA) on a Simoa HD‐1 Analyzer instrument, according to the manufacturer's instructions. Plasma samples were diluted at a ratio of 1:4, and the detection range of the kit was 0–1000 pg/mL. Samples were anonymized by assignment of random numbers to replace identifying information, and measurement was performed by technicians with no knowledge of the experiment.

### Statistics

Categorical variables were described by counts and percentages, while continuous and ordinal variables were described by median and interquartile ranges. Continuous variables that followed normal distributions were compared using *t* test, and variables that violated the assumptions of normality were analyzed by the Mann–Whitney *U* test. Categorical variables were compared by chi‐square test and Fisher exact test. Abnormal GFAP values were defined as pGFAP concentrations greater than 132.48 pg/mL (one‐sided confidence interval, mean value +1.64 standard deviation of HCs). Multiple regression analysis was used to eliminate potentially confounding factors (sex, age, age at onset, different treatments) that may influence pGFAP levels. Receiver operating characteristic (ROC) curve analyses were performed to assess diagnostic sensitivity and specificity of pGFAP detection. We used a linear regression model to explore associations between pGFAP concentration and disease severity. Statistical analysis was performed using SPSS version 26.0 software (SPSS, Inc.) and GraphPad Prism version 8.3 software (GraphPad, Inc.). *P* < 0.05 was considered statistically significant.

## Results

Demographic and clinical data for all patients are listed in Table 1. On average, patients with WD‐HM were younger than patients with WD‐NM and HCs [20.5 (16.3–29.0) vs 29.5 (24.0–37.0) vs 35.0 (25.0–44.5); *P* < 0.05]. The groups showed no significant difference in sex ratio.

**TABLE 1:** Demographic and clinical characteristics

Characteristics	WD‐NM (n = 74)	WD‐HM (n = 20)	*P* Value
Sex, n (%)			0.540^a^
Female	39 (53)	9 (45)	
Male	35 (47)	11 (55)	
Age, median (IQR), yr	29.5 (24.0–37.0)	20.5 (16.3–29.0)	0.002^b^
Age at onset, median (IQR), yr	19.0 (16.0–27.0)	11.0 (7.0–22.5)	0.001^b^
Disease duration, median (IQR), yr	8.0 (4.0–13.3)	8.5 (3.8–13.0)	0.767^b^
UWDRS‐F, median (IQR)	1 (4–9)	0 (0–0)	<0.001^b^
UWDRS‐N, median (IQR)	14 (7–33)	0 (0–0)	<0.001^b^
UWDRS‐P, median (IQR)	4 (2–8)	0 (0–1)	<0.001^b^
Chelating agent treatment, n (%)			0.003^c^
No	5 (7)	7 (35)	
Yes	68 (93)	13 (65)	
Zinc salts treatment, n (%)			1^c^
No	7 (10)	1 (5)	
Yes	66 (90)	19 (95)	
Abnormal MRI, n (%)			<0.001^c^
No	2 (4)	6 (75)	
Yes	52 (96)	2 (25)	

^a^ Pearson chi‐square test.

^b^ Mann–Whitney *U* test.

^c^ Fisher exact test.

WD‐NM, Wilson disease with neurological manifestations; WD‐HM, Wilson disease with hepatic manifestations; IQR, interquartile range; UWDRS‐F, Unified Wilson Disease Rating Scale, functional impairment; UWDRS‐N, Unified Wilson Disease Rating Scale, neurological examination; UWDRS‐P, Unified Wilson Disease Rating Scale, psychiatric assessment.

**FIG. 1:**
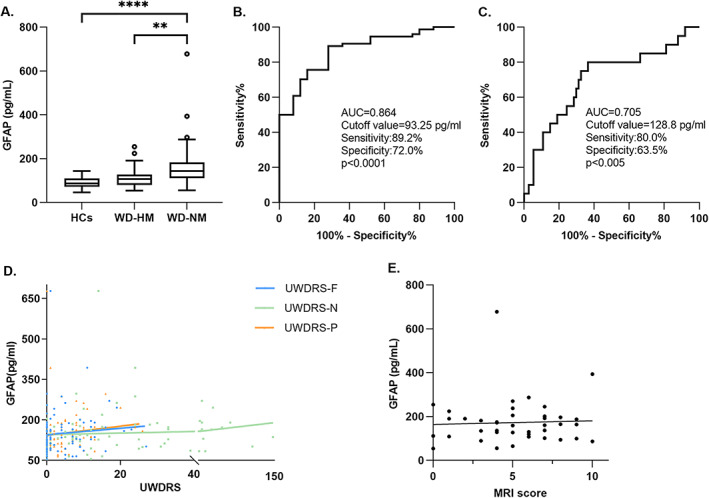
Plasma glial fibrillary acidic protein (pGFAP) levels in different groups and its correlation with disease severity. (**A**) pGFAP levels in healthy control subjects (HCs), Wilson disease with hepatic manifestations (WD‐HM), and Wilson disease with neurological manifestations (WD‐NM) groups shown in box and whisker (Tukey) plots (**P < 0.01, ****P < 0.0001). Receiver operating characteristic curve analyses for differentiating patients with WD‐NM from (**B**) HCs and (**C**) patients with WD‐HM. (**D**) Correlation between neurological symptom severity at sampling and pGFAP levels in WD‐NM. (**E**) Correlation analysis between brain parenchyma damage severity and pGFAP levels. AUC, area under the curve; UWDRS‐F, Unified Wilson Disease Rating Scale, functional impairment; UWDRS‐N, Unified Wilson Disease Rating Scale, neurological examination; UWDRS‐P, Unified Wilson Disease Rating Scale, psychiatric assessment.

In patients with WD‐NM, pGFAP levels [143.87 (110.87–183.05) pg/mL] were greater than in patients with WD‐HM [107.50 (80.07–128.00) pg/mL; *P* = 0.0045, Mann–Whitney *U* test] and HCs [86.85 (71.84–109.47) pg/mL; *P* < 0.0001, Mann–Whitney *U* test] (Fig. 1A). After adjusting for age and sex with logistic regression analysis, no significant differences were found between patients with WD‐HM and HCs [odds ratio (OR), 1.032; *P* = 0.978]. Logistic regression analysis also revealed that phenotype was the independent influencing factor that affected pGFAP levels between WD‐NM and WD‐HM (OR, 6.249; *P* = 0.007) after adjusting for sex, age, age at onset, and different treatments. To assess the ability of pGFAP to distinguish between patients with WD‐NM and HCs, we performed ROC curve analysis, which showed that a pGFAP cutoff value of 93.25 pg/mL resulted in 89.2% sensitivity and 72.0% specificity, with an area under the curve of 0.864 (Fig. 1B). The ROC curve revealed that a pGFAP cutoff value of 128.8 pg/mL also provided sufficient sensitivity (80.0%) and specificity (63.5%) to differentiate WD‐NM from WD‐HM, with an area under the curve of 0.705 (Fig. 1C).

Using a linear regression model to examine the relationship between pGFAP levels and neurological symptom severity in patients, we found that pGFAP levels showed no relationship with UWDRS‐F subscore (β = 0.095; *P* = 0.954), UWDRS‐N subscore (β = 0.051; *P* = 0.896), or UWDRS‐P subscore (β = 0.825; *P* = 0.650) (Fig. 1D). Nor was any significant correlation observed between GFAP levels and MRI score in WD (β = 1.649; *P* = 0.785) (Fig. 1E).

In addition, we observed the clinical characteristics of two patients with WD‐HM who had abnormal levels of pGFAP. Case 1, an 8‐year‐old boy, was found to exhibit abnormal liver function during a medical checkup at the age of 1 year. From that time until present, he was kept on a low‐copper diet with daily supplements of zinc salts and regular follow‐up examinations. His MRI was normal, ultrasound showed echogenic liver parenchyma, and 24‐hour urinary copper level was 82.03 μg during the study. Case 2, a 29‐year‐old woman, initially sought medical advice for edema of both legs at age 13 years and took zinc salts daily for an extended period. Her MRI was normal, ultrasound showed echogenicity of liver parenchyma with splenomegaly, and her 24‐hour urinary copper level was 69.43 μg during the study.

## Discussion

Previous studies have reported that pGFAP concentration is related to brain injury.^18^, ^19^, ^20^ In light of these findings, we performed this study to investigate whether pGFAP concentration could function as a marker for brain damage associated with WD. Interestingly, the results of this study demonstrated that pGFAP levels were obviously elevated in patients with WD‐NM compared with those in patients with WD‐HM and HCs. After multiple regression analyses of age, age at onset, chelating agent treatment, and phenotype, we found that phenotype was the independent factor influencing pGFAP levels. According to our results, pGFAP levels may be a sensitive diagnostic biomarker for distinguishing WD‐NM from WD‐HM and HCs, which can potentially reflect the presence of brain damage in patients with WD.

We also explored the correlation between pGFAP levels and disease severity, that is, clinical symptoms and MRI lesions. Our results showed that pGFAP levels were not correlated with disease severity. Two reasons at least partially explain this result. Disease severity is a function of long‐term damage accumulation in WD, which is a cumulative value. Thus, pGFAP concentration at a specific time point may not reflect general brain damage. Moreover, a nonlinear natural history of CNS injury progression in WD may contribute to fluctuations in pGFAP concentration that prevent a continuous correlation between disease severity and pGFAP.

Given the rarity of the longitudinal data in this study, we could not definitively determine whether pGFAP is consistent and reliable as a biomarker for disease severity, disease activity, and treatment response on neurological injury in WD. The cohort size is small, and the sensitivity and specificity of the ROC analysis of GFAP levels are not very robust, so the results of this study should be interpreted with caution. Nevertheless, the results of this study may urge further evaluation by large‐scale clinical trials to explore biomarkers for distinguishing WD‐NM from WD‐HM.

## Author Roles

Y.F., N.W., and W.‐J.C. conceived the study concept and design. J.L., Y.Z., Yi Lin, X.‐H.L., and W.‐H.L. acquired the data. Laboratory determination was done by W.Z. Y.F. and Ying Liu. analyzed the data. J.L. and Q.W. performed the statistical analysis. Y.F. and J.L. drafted the manuscript.

## Financial Disclosures

All authors declare that there are no additional disclosures to report.

## Ethical approval

The study was approved by the First Affiliated Hospital of Fujian Medical University institutional review boards and ethics committee.
